# Hygienic practices and associated factors in slaughterhouses and meat retail shops in Hawasa City, Ethiopia

**DOI:** 10.1371/journal.pone.0336784

**Published:** 2025-11-13

**Authors:** Lakew Desta Zewude, Embialle Mengistie, Amanuel Ejeso

**Affiliations:** 1 Department of Environmental Health, School of Public Health, College of Health Science and Medicine, Arbaminch University, Arbaminch, Ethiopia; 2 Departments of Environmental Health, College of Health Science and Medicine, Hawassa University, Hawassa, Ethiopia; Xinjiang Medical University Affiliated First Hospital, CHINA

## Abstract

**Background:**

In developing countries, animals are often slaughtered and dressed in unsanitary settings, thus endangering the microbiological quality and safety of the meat obtained. In addition, slaughterhouses and retail outlets are the regions most vulnerable to cross-contamination with meat. As a result, the objective of this study was to evaluate slaughterhouse and meat retail shop hygiene practices and to determine the causes of unsanitary meat handling in slaughterhouses and retail shops.

**Methods:**

165 butcher men from a municipal slaughterhouse and every retail meat store in Hawassa city participated in this investigation, which was carried out using an institution-based cross-sectional study design between March 22 and May 30, 2022. Data was collected through interviewer-administered standardized questionnaires and an observation checklist. Data was entered using Epidata software version 3.1.1, and it was exported for further analysis using SPSS version 25. A cutoff point of P.V < 0.25 was employed for the bivariate model to control possible confounders. A binary logistic regression analysis was conducted to identify independent factors, the findings were displayed as the 95% confidence interval for the crude odds ratio and adjusted odds ratio. The level of significance was assessed using cutoff points of P < 0.05.

**Results:**

The prevalence of good hygienic practices was 25.5% [95% CI: (18.2–32.1)]. Attitude (fair attitude) [AOR = 0.157; 95% CI: (0.037, 0.659)], cleanness and disinfection of waste container status [AOR = 12.64; 95% CI: (3.936–40.562)], regular supervision by health offices [AOR = 0.176; 95% CI: (0.35–0.894)], health and safety training [AOR = 3.46; 95% CI: (1.054–11.344)], and Removal of personal items during meat processing [AOR = 0.036; 95% CI: (0.008–0.150)] were factors that were significantly associated with poor hygienic practices.

**Conclusion:**

The prevalence of good hygienic practices was low. After adjusting for possible confounding factors, attitudes, cleanness and disinfection of waste containers, regular supervision by health offices, health and safety training and Removal of personal items during meat processing (behavioral factor) were significantly associated with hygienic practices. These findings could call for appropriate prevention strategies based on cognitive domains and practice.

## Introduction

A slaughterhouse is a unique facility that receives, holds, slaughters, and inspects meat animals and meat products before they are released for public consumption [1]. The examination of a live animal before slaughter (antemortem examination), slaughtering, evisceration, carcass inspection (postmortem inspection), and waste disposal are all part of proper slaughterhouse operations, and all of these steps are necessary for the delivery of safe meat and the surveillance of animal diseases, particularly those that are of public health concern [2]. Basic operating and environmental conditions of appropriate sanitary and hygienic practices, as well as standard operating procedures, are required in slaughterhouse practices for the production of safe meat [3].

Foodborne infections can spread from cattle slaughterhouses through the processing and distribution continuum, including retail outlets, and eventually reach people. As a result, effective hygienic measures at slaughterhouses, throughout distribution to and storage at retail stores, as well as during sales, are critical for ensuring the quality and safety of meat to protect public health [4,5]. Inadequate facilities and poor animal handling at slaughterhouses exacerbate microbial contamination of beef, potentially resulting in the spread of foodborne diseases to people [6,7]. There is a need to reduce food risk in the meat supply chain, which must begin at the farm level, slaughterhouses, butcher shops, and retail outlets; however, slaughterhouses and retail outlets are the regions most vulnerable to cross-contamination of meat.

Globally, approximately 75% of new communicable diseases that have affected humans in the last ten years have been caused by pathogens originating from animals or animal products [8]. Many of these novel human diseases are known as zoonotic diseases, and they are linked to the handling of sick domestic and wild animals, as well as slaughtering, meat cutting, retailing, and processing [8]. Foodborne disease outbreaks remain a major global health challenge, and cross-contamination from raw meat due to inadequate handling is a major source of foodborne disease epidemics in poorer nations [9].

According to the World Health Organization (WHO), every year, nearly 1 in 10 people become ill, and 420,000 people die because of eating food infected by microbes [10]. Moreover, approximately 600 million foodborne illnesses and 420,000 fatalities occur each year as a result of inadequate food handling practices, with meat-related hazards accounting for a significant fraction (approximately 56%) of the total [11]. The global burden of foodborne diseases caused by all animal source foods and beef is estimated to be 168 and 10 disability-adjusted life years per 100,000, respectively [12]. Diet high in red meat and processed meat was an apparent characteristic of Western diet pattern; however, previous systematic reviews had demonstrated positive associations between red meat consumption and all-cause mortality and cardiovascular mortality and stroke [13–15]

Most of meat-borne bacterial outbreaks are caused by contamination in the supply chain because of inadequate handling techniques [16]. Food-producing animals are a key source of many foodborne pathogens and can lead to meat contamination, which can cause foodborne diarrheal infections in humans [17,18]. Animals are often slaughtered and dressed in unsanitary settings in developing countries, thus endangering the microbiological quality and safety of the meat obtained from the animals [19,20]. For example, fecal carriage of *E. coli* O157:H7 in animals increases the risk of these bacteria entering the food chain through fecal contamination of meat with intestinal contents during slaughter. The infection can be found on the skin or in the feces of the animal during slaughter, and it can be transferred to the carcass during evisceration or skin removal. As a result, inadequate slaughter operations, especially poor hygienic measures during slaughter, transport, and display of meat, play a significant role in increasing meat contamination [21].

Meat quality and safety are harmed by butcheries’ informal techniques of meat handling and marketing [20–24], a problem that is shared by Ethiopia’s similar socioeconomic situation. Food is traditionally sold and displayed in open shops without sufficient security [25], and the cold-chain process [26] of hanging raw meat on the hooks and oﬀering it for consumption to consumers is followed in butcheries [27]. Several studies have investigated the presence of pathogens throughout the meat supply chain [20,21,28–31]. On the other hand, contamination at specific levels, such as slaughterhouses [19,32–34] and retail shops [34–36], has been detected in different countries, including Ethiopia. According to a study conducted in Addis Ababa, Ethiopia, beef contamination occurs during the transition from abattoirs to butcher shops, with abattoir personnel being the most common source of contamination [20,26].

There are approximately 300 small slaughterhouses in Ethiopia that supply meat for local consumption, each with a different capacity and facility, but all have poor hygienic standards [25,37]. Despite growing prices, demand for meat products in Ethiopia has increased considerably, from 7.0 kg in 2000 to 8.5 kg in 2009 per capita [38]. Owing to traditional and cultural norms, the demand for ready-to-eat items such as raw beef has increased in Ethiopian cities and towns, such as Hawassa city [38]. Although several studies have assessed food safety knowledge, attitude, practice, and meat contamination in Ethiopia, there remains a significant gap in understanding the specific factors that influence hygienic practices at slaughterhouses and meat retail shops (reference). Existing research primarily focuses on microbial contamination levels and general hygiene awareness, with limited emphasis on the behavioral, institutional, and environmental factors that directly shape hygienic handling in slaughterhouses and meat retail shops [39–46]. Additionally, most available studies are localized and do not provide a comprehensive national or regional perspective, leaving key determinants such as training,regulatory enforcement, infrastructure, and resource availability underexplored [39,47–49]. Understanding these factors is crucial because unsafe handling practices remain a leading cause of foodborne diseases in Ethiopia, contributing to a substantial public health burden and economic losses [11,50]. As a result, this study is essential to fill the evidence gap regarding structural and behavioral determinants rather than merely describing contamination prevalence. Thus, the objective of this study was to assess hygienic practices and associated factors at cattle slaughterhouses and meat retail shops in Hawassa City.

## Methods and materials

### Study design and study period

An institutional-based cross-sectional study was conducted from March 22- May 30, 2022.

### Study setting

This study was conducted at a local municipal cattle slaughterhouse and all retail shops selling meat found in Hawassa city. The city is in the Sidama region, 275 km south of Addis Ababa, Ethiopia [51,52]. According to the Hawassa online data from 2021, the projected current total human population of Hawassa city is estimated to be 329,734. A total of 59.71% of the population were Protestants, 26.99% practiced Ethiopian Orthodox Christianity, 8.14% were Muslim, and 3.78% embraced Catholicism [52,53]. The slaughterhouse is administered by the city municipality. Retail shop owners bought cattles directly from open markets to be butchered at the slaughterhouses. It is operated by one junior veterinarian and three assistant meat inspectors [54]. According to the information obtained from the Hawassa City Trade and Industry Office, there was 1 (one) municipality slaughterhouse that provides a slaughtering service to provide raw meat for a total of 141 butcher shops. Currently, slaughterhouses usually slaughter 70–90 cattle per day.

### Participants

#### Source population.

The source population to participate in this study was all butchers in the slaughterhouse and all meat retail shop butchers in Hawassa city, which include meat processors, meat sellers and sanitary officials.

#### Study population.

The study populations were participated in this study was the butchers in the slaughterhouse and selected meat retail shop butchers in the selected slaughterhouse and retail shop of the Hawassa city administration.

### Variables

#### Dependent variables.

Hygienic practices in slaughterhouses and meat retail shops.

#### Independent variable.

Sociodemography (age, sex, educational status, work experience, occupation), water supply, sanitation and hygiene facilities (solid & liquid waste disposal, hand washing facility, housing conditions that prevent insects and rodents), regulatory authority, contact surface, work experience, source of meat, knowledge of food safety and health, attitudes toward food safety and health, storage environment and conditions, and personal hygiene.

### Eligibility criteria

#### Inclusion criteria.

The participants who were meat retailers in retail shops/meat processors in slaughterhouses were included in the study. The criterion for participation in the study was a volunteraily to participate in the study and the ability to respond to the questions to the end of the interview.

#### Exclusion criteria.

Meat retailers in retail shops/meat processors who did not provide informed consent and who had less than six months of experience were excluded from the study.

### Data source

#### Sampling technique and sample size determination.

The target site of for this study was Hawassa city municipality Slaughterhouse and all meat retailshops. The target participants were the slaughterhouse buthers men and buthers in meat retail shope. This study used the census sampling method. The total number of meat processers or butchers working in the slaughterhouse was 24, and the total number of meat retailers in retail shops was 141 in a total of 141 meat retail shops found in Hawassa city. The sampling technique was employed for this study was purposive sampling techenique. By the sampling technique all study population were participated based on inclusion criteria. The sample size for the questionnaire survey was all 24 the slaughterhouse meat processers (butchers) and all 141 the meat retailers (butchers) from retail shops were included. Then, the final sample size becomes 165.

#### Data collection tools.

Data were collected using a pretested structured, interviewer-administered questioner to assess hygienic meat handling practices at slaughterhouses and meat retail shops. The questionnaires and checklist were adapted from similar previous studies conducted in Uganda and Ethiopia [55,56].

#### Data collection procedure.

Data were collected on the hygienic practices of slaughterhouse and meat retail shop workers’ socio-demography, meat shop profile, butcher profile, awareness of meat hygiene, knowledge of the health effects of inadequate meat handling, wearing of Personal Protective Equipment (PPE), cleanliness of equipment, regular hand washing and disinfection practices of hands and byproduct disposal. In addition, observations were made of personal hygienic practices, meat storage conditions, sanitation and hygiene facilities, the availability of clean tap water, the floor and processing tools and prevailing environmental conditions via observational checklists. The Personal Protective Equipment (PPE) assessed included the use of an apron, gloves, footwear, a mask, and a head cap. Three environmental health specialists with a Bachelor of Science (BSc) in Environmental Health collected the data. All employees in the slaughterhouses (municipal = 24) and one employee from each of the retail shops (n = 141) engaged in meat handling activities were included in the study.

In the hygiene practices part, the question comprises the issues of personal hygiene, hand washing practices, waste management and cross contamination. This section has 27 questions with two possible responses: “yes” and “no”. Each correct practice reported a score of one (1) point.

#### Sample collection for swab test procedure.

For this study all 141 meat retail shop sampling unit were selected for swab test purposively. These units were selected for swab sample collection. Samples were collected at the slaughterhouse included; swabs from Slaughterhouse environments (evisceration’s knife (n = 10) and hands (n = 10), cutting boards (n = 10), and meat processers’ protective clothes (n = 10)), whereas from the retail shops, swab samples of knife (n = 141), butcher’s hand (n = 141), cutting board (n = 141) and protective cloths (n = 141) was collected over a period of 8 weeks. Sampling points in the slaughterhouse and at meat retail shops were selected according to the problem areas in the process (after dehiding, after evisceration and after display for sale). To collect the data, one visit to the municipal abattoir and all officially registered butcher shops (n = 141) was made per week for a consecutive 8 weeks.

#### Environmental samples collection.

A total of 604 swab samples (40 from the slaughterhouse and 564 from meat retail shops) were collected from the hands of meat handlers, their protective clothing, knives and chopping boards after eviscerations at slaughterhouse and before the beginning of the operation at the meat retail shops (which are triplicate samples). The swab samples were taken from 15–20 cm^2^ of meat contacting surface using sterile, buffered peptone water moistened cotton swabs [43]. All the collected samples was labeled, packaged in sterile, separate containers and carried to the Environmental Health laboratory of Hawassa University in a cold box immediately after collection for processing.

#### Isolation and identification process for *E.Coli.*

***Enrichment Processes*:** To determine the sanitary status of the slaughterhouse and retail shops it is helpful to detect sanitary indicator bacteria (E.coli) from swab samples which are collected from slaughterhouse and meat retail shops. First all swab samples from the slaughterhouse and retail shops were homogenized in 9 mL of modified tryptone soya broth (mTSB) for each and were cultured at 37°C for 24 hours [57]

***Identification of the Isolates*:** The overnight broth cultures were spread onto MacConkey agar [57]. The inoculated plates were incubated at 37°C for 24 hours. The isolated colonies first were screened by colony morphology, color production (lactose fermenting red/pink colonies) and identified as *E. coli* by relevant biochemical tests, such as indole test.

#### Determination of microbial load.

Appropriate plates containing distinct microbial colonies was selected and counted using a colony counter. Then, the microbial load will determined using the standard formula as follows [36]


$$N=\frac{\text{n}}{s\;\times \;d^{\prime }}$$

Where N = total number of bacteria (cfu) per ml of the sample, n = average number of bacterial colonies, from different dilutions (10^−1^ − 10^−3^) in Petri dish that contained 30–300 colonies, s = volume of sample for plating, and d = dilution factor of the specimen/food sample.

### Data quality management

Data collectors and supervisors received training. Before the actual data collection, the study questionnaires were translated from English versions to Amharic versions and tested on 10 randomly selected meat retail shops. Then the responses filled in on the study questionnaires were translated from the Amharic versions to the English versions to check consistency. This allowed the data collector to become more familiar with the content of the questionnaire. Daily questionnaire checks, meticulous coding of completed questionnaires, and double data entry for validation were made possible. For swab test, the sterility of cotton sticks and bottles was tested, as well as the presence of alkaline or acidic residue on glassware. Transported samples were kept at 10°C until chilled or tested. The sterility of the media, buffered dilution and rinse water, pipettes, flasks, and petri dishes, as well as the equipment used throughout the inoculation operation, was all tested. Positive and negative control organisms were used to test the performance and sterility of the media. No media that failed media quality control protocols was used. Each analyst’s ability to reliably count bacterial colonies on solid media was tested. The discrepancies in count between analysts were not more than 10%. Biochemical reactions and growth characteristics are used to identify bacterial colonies isolated from positive samples. Temperatures in incubators and refrigerators were regularly checked using calibrated thermometers to ensure that the required temperatures for sample storage and bacterial growth were met. Every day of laboratory use and operation, the required temperature ranges for all equipment were documented on temperature charts at least once. Every time the autoclave was used, the temperature was verified.

### Statistical methods

Data entry was performed via Epidata software version 3.1.1, and the data were exported to Statistical Package for the Social Sciences (SPSS) version 25 for further analysis. Descriptive statistics included the means, medians, standard deviations, and ranges for continuous data and percentages and frequency tables for categorical data. For the bivariate model, P.V < 0.25 was used as a cutoff point in the subsequent model to control possible confounders. Model fitness was checked by using Nagelkerke R^2^ the value was 0.584 which means the model explains 58.4% of the variation in the outcome and the Hosmer and Lemshow test [58], which yields values greater than 0.05 the reported value was 0.994 which means the model fits the data well or good fit this indicate there was no significant difference between the observed and predicted outcome, and multicollinearity was checked via tolerance>0.2 [59] and the reported value lies 0.690–0.929 for all indpendente candidiadate variables in binary logistic regression and variance inflation factor (VIF) <5 [59] the reported value lies 1–1.448 for all indpendent candidiadate variables in binary logistic regression which means acceptaple or there was no any multicollinerity issue in the independent variable. A binary logistic regression analysis was carried out to identify independently associated factors. Finally, the results are presented as the crude odds ratio (COR) and adjusted odds ratio (AOR) with 95% confidence interval (CI). P values less than 0.05 were used as cutoff points to determine the level of significance. From the swab test data entered in Excel, the maximum bacterial count, mean, and standard deviation were used to describe the result.

### Operational definition

**Hygienic practices:** overall hygienic practices in slaughterhouses and meat retail shops. These include individual hygienic practices, waste management, and insect and rodent management, which ensure the safety of meat handling.

**Slaughterhouse hygiene:** Where animals are slaughtered, how animals are slaughtered and dressed, and waste disposal in the slaughterhouse were considered to constitute knowledge about slaughterhouse operations.

**Good hygiene practices:** Respondents whose answers/scores were ≥ 70% or 19 or more out of 27 hygienic practices “yes” or “no” questions, was defined as “good hygienic practices” [56].

**Poor hygienic practices:** Respondents whose answers/scores were < 70% or less than 19 out of 27 hygienic practices “yes” or “no” questions, was defined as “poor hygienic practices” [56].

**Knowledge of food safety and health:** The questionnaire concerning Knowledge on food safety and health comprised 12 close-ended questions with two possible answers: “Yes” and “No”. These questions specifically dealt with respondents’ knowledge of hand hygiene, cross contamination, food-borne diseases, temperature control and hygienic practices. Food-handlers who obtained total scores ≤8 points were considered to have “**poor**” knowledge, and food-handlers who obtained total scores ≥9 points (≥68% accuracy) were considered to have “**good**” knowledge of food safety [60].

**Attitude:** Twelve positive statements concerning the opinion attitudes of meat handlers regarding meat hygiene were presented. The rating scale was measured as follows: positive statements with strongly agree, agree, indifferent, disagree, and strongly disagree were given scores of 5, 4, 3, 2 and 1, respectively. The scores ranged from 1 to 60. Each score was summed, and the means of each score were calculated. The overall mean score was 45.23 ± 6.44 STD (Standard Deviation).

This was classified into three categories:

- ≥ mean score = **good attitude** (45–60),
- 1 STD < mean score = **fair attitude** (44–39) and
- **poor attitudes** (12–38) [23].

## Results

### Sociodemographic characteristics

A total of 165 meat handlers participated in the research, and the response rate was 100%. Almost all the respondents (164, 99.4%) were men, and half of them (50.3%) were between the ages of 26 and 34. The mean age of the respondents was 29.33 years ± 5.97 years. The majority of the 100 (60.6%) respondents had completed primary school. More than half (88, 53.3%) of the respondents were married. The respondents’ religions were 55.8% orthodox, 43.6% protestant, and 0.6% Muslim. Below half (34.5%) of the meat retail shop owners had an elementary education, whereas the remaining 108 (65.5%) had secondary or higher education (Table 1).

**Table 1:** Sociodemographic characteristics of respondents in slaughterhouse and meat retail shops in Hawassa city, Ethiopia.

Respondent information	Category (n = 165)	N (%)
Respondent gender	Male	164(99.4%)
	Female	1(0.6%)
Age (in years) of respondents		
	≤ 25	48(29.1%)
	26-34	83(50.3%)
	≥ 35	34(20.6%)
Educational level of respondents		
	no formal education	13(7.9%)
	Primary	100(60.6%)
	secondary and above	52(31.5%)
Marital status of respondent		
	Married	88(53.3%)
	Single	77(46.7%)
religion status of respondent		
	Protestant	72(43.6%)
	Orthodox	92(55.8%)
	Muslim	1(0.6%)
Educational level of meat retail shop owner		
	Primary	57(34.5%)
	secondary and above	108(65.5%)
Job experience	1-4 years	78(47.1%)
	5-8years	68(41.2%)
	≥9 years	19(11.5%)
Monthly salary	≤1000	11 (6.7%)
	1001-2500	97 (58.8%)
	2501-3500	50 (30.3%)
	≥3501	7 (4.2%)
Employment status	Daily	21(12.70%)
	Contract	29(17.60%)
	Permanent	115(69.70%)

### Training and supervision

More than half or 93 (56%) of the study participants had never received any health or safety training on meat handling procedures. On a regular basis, health offices supervised 144 (87.3%) of the meat retail shops. In addition to their meat-handling work, 31.5% of butchers had a second job, and 52 (31.5%) of the study participants did not have regular medical checkups for general health.

### Prevalence of hygienic practice

This study found that the prevalence of good hygienic practices in slaughterhouses and meat retail shops was 25.5% (95% CI: 18.2–32.1).

Almost all 165 (100%) of the butcher men used to wash their hands with soap and water after visiting the toilet. Approximately 5 (3.0%) butchers do not wash their hands before slaughtering. Above two-thirds, 138 (84%) butcher men always dispose of waste such as animal dung, horns, and scraps of tissue from their workplace. A total of 156 (94.5%) butcher men did not wash their hands before and after handling meat. In addition, 148 (89.7) butchers were not washing their hands after handling waste/garbage. Sixty-one (37%) of the study subjects did not wash their hands after smoking, sneezing, or coughing. Among all the meat handlers, only 73 (44.2%) individuals wore an apron while working, but the majority did not.

Approximately 54 (32.7%) butchers were not washing their aprons after each day’s work. Additionally, 51 (30.9%) of the respondents were not wearing a hairnet or a cap while working. A high proportion of 151 (91.5%) butcher men did not properly clean the meat storage area before storing new products. Only 75 (45.5%) of the respondents used disinfectants when washing. In line with this, 152 (92.1%) butchers did not replace knives or sterilizers after each meat processing. Only 24 (14.5%) butcher men removed their working equipment, such as an apron, when using toilets. Almost half of the 87 (52.7%) butcher men did not remove their personal items, such as rings and watches, while processing meat. In addition, 32 (19.4%) of the respondents said that they handled meat when it was ill. In line with this, 22 (13.3%) of the respondents said they handled meat when they had wounds, bruises, or injuries to their hands. Almost half 80 (48.5%) of the units’ floors, walls, roofs, doors, and window openings were not in a good state of repair, with gaps or spaces that could not control the pest. However, in 62 (37.6%) of the study subjects, the meat was not kept in pest-proof containers. Approximately 92 (55.8%) of the unit’s waste containers were not regularly cleaned or disinfected (Table 2).

**Table 2:** Meat handlers’ hygienic practices in Slaughterhouse and meat retail shops in Hawassa city, Ethiopia, (n = 165).

Variables	Response (n = 165)	
	Yes n (%)	No n (%)
Wash their hands with soap and water after toilet	165(100.0%)	0.0%
Wash their hands before slaughtering activities	160(97.0%)	5(3.0%)
Always dispose waste such as feces, horns and scraps tissue	138(84%)	27(16%)
smoke inside meat processing areas	30 (18.2%)	135 (81.8)
wash their hands properly before or after using gloves	160(97%)	5(3%)
Wash their hands before and after handling meat	156(94.5%)	9(5.5%)
Wash their hands after handling waste/garbage	17(10.3%)	148(89.7%)
Wash their hand after smoking, sneezing or coughing	104(63.0%)	61(37.0%)
Wear an apron while working	73(44.2%)	92(55.8%)
Wash their aprons after each day’s work	111(67.3%)	54(32.7%)
wear a mask while working	148(89.7%)	17(10.3%)
Wear a hairnet or a cap while working	114(69.1%)	51(30.9%)
Properly clean the meat storage area before storing new products	14(8.5%)	151(91.5%)
Use the disinfectant when washing service utensils	75(45.5%)	90(54.5%)
Replace knives or sterilizes them after each meat processing	13(7.9%)	152(92.1%)
Remove work equipment when using toilets	24(14.5%)	141(85.5%)
Remove personal stuffs such as rings, watch while processing meat	78(47.3%)	87(52.7%)
Handle meat when they are ill	133(80.6%)	32(19.4%)
Handle meat when wounds, bruises or injuries on their hands	143(86.7%)	22(13.3%)
The ﬂoors, walls, roof, doors, and window openings in a good state of repair with no gaps or spaces can control the pest	85(51.5%)	80(48.5%)
Keeping the meat in pest-proof containers	103(62.4%)	62(37.6%)
Inedible parts is placed in containers with suitably	25(15.2%)	140(84.8%)
Use of waste containers but without lids	60(36.4%)	105(63.6%)
Waste containers regularly cleaned and disinfected	73(44.2%)	92(55.8%)
Presence of separated room for waste containers	29(17.6%)	136(82.4%)
Presence of waste control or management plan	29(17.6%)	136(82.4%)
Presence of rodent/fly/insect trap	19(11.5%)	146(88.5%)
**Over all hygienic practice result**	**Good hygienic practice**	**42(25.5%)**
	**Poor hygienic practice**	**123(74.5%)**

### Respondents’ knowledge of food safety and health

Only 79 meat handlers were aware that “improper handling of meat could pose health hazards to consumers” (47.9%).Sixty three (38.2%) respondents were aware that “washing hands before and during meat processing reduces contamination”. The meat handlers’ awareness that “insects and pests could be a source of contamination to raw meat” was 69 (41.8%), but the majority were not aware. Almost half of the study subjects (84, 50.9%) were not aware of “cross-contamination” (i.e., when microorganisms from contaminated meat are transferred by the meat handler’s hands or utensils to another”). Half of the meat handlers (74, 44.8%) were not aware of “the ideal place to store raw meat is in the refrigerator.” Meat handlers were aware that “the correct temperature for storing perishable foods is 8–10°C” was only 114 (69.1%). More than two-thirds of the meat handlers (133, 80.6%) were unaware that “people with open skin injuries are not permitted to handle meat.” Meat handlers who were aware that “the health status of workers should be evaluated before employment” numbered only 17 (10.3%). The majority of the 105 (63.6%) did not have health certificates. Half (53.9%) of the butcher men had poor knowledge of food safety and health (Table 3).

**Table 3:** Knowledge status of respondents on food safety and health in slaughterhouse and meat retail shops in Hawassa city, Ethiopia.

Variable	Response (n = 165)	
	Yes n (%)	No n (%)
Improper handling of meat could pose health hazards to consumers?	79(47.9%)	86(52.1%)
Washing hands before and during meat processing reduces contamination?	63(38.2%)	102(61.8%)
cleaning of knives and hooks reducing the risk of meat contamination	67(40.6%)	98(59.4%)
washing of working surfaces and tools are important for safety of meat	72(43.6%)	93(56.4%)
Insects and pests could be a source of contamination to raw meat?	69(41.8%)	96(58.2%)
Cross contamination is when microorganisms from a contaminated meat are transferred by the meat handler’s hands or utensils to another?	81(49.1%)	84(50.9%)
Is the ideal place to store raw meat is in the refrigerator	91(55.2%)	74(44.8%)
The correct temperature for storing perishable foods is 810^0^C	114(69.1%)	51(30.9%)
People with open skin injury is not allowed to handle meat	32(19.4%)	133(80.6%)
The health status of workers should be evaluated before employment	17(10.3%)	148(89.7%)
haveing food safety training can reduce meat containation	55(33.3%)	110(66.7%)
Do you have Health certificate	60(36.4%)	105(63.6%)
**Knowledge rank**	**Poor knowledge**	89(53.9%)
	**Good knowledge**	76(46.1%)

### Attitudes toward food safety and health

In this study, two-thirds of the respondents 125 (75.8%) strongly agreed that “meat handlers with wounds or injuries on their hands they shoudn’t handle meat”. However, 21 (12.7%) of the respondents disagreed that “using watches and rings increases the risk of meat contamination”, and 27 (16.4%) of the meat handlers strongly disagreed that “handwashing before handling meat reduces the risk of contamination” (Table 4). Regular training could improve meat safety and hygiene practices; 38 (23%) of the study meat handlers were neutral. Eighteen (10.9%) of the respondents strongly disagreed that “safe meat handling to avoid contamination is part of the meat handler’s job.” A total of 37 (22.4%) meat handlers strongly disagreed that “surfaces and equipment should be cleaned before being reused for meat processing.” moreover, 47 (28.5%) of the meat handlers were neutral on “whether knives, hooks, and cutting boards could be a source of food contamination”. Approximately 11 (6.7%) of the respondents strongly disagreed with the idea that “sneezing without covering our noses or mouths could contaminate the meat”, and 8.5% of the respondents disagreed with it, whereas 38 (23%) of the respondents were neutral (Table 5). In addition, 15 (9.1%) meat handlers strongly disagreed that “wearing protective clothing and shoes could help improve hygiene practices”, and 26 (15.8%) respondents strongly disagreed that “putting on a hair cover on the head is a good practice in the food industry”. In this study, 30.9% (57.6%) of the meat handlers had good attitudes toward food safety, 30.9% had fair attitudes, and 11.5% had poor attitudes toward food safety (Table 4).

**Table 4:** Attitude status of respondents’ responses to questions concerning food safety and health in slaughterhouse and meat retail shops in Hawassa city, Ethiopia- (n = 165).

Question items	strongly agree	Agree	Neutral	Disagree	Strongly disagree
Meat handlers with wounds or injuries on their hands they shoudn’t handle meat	125 (75.8%)	5 (3.0%)	2 (1.2%)	4 (2.4%)	29 (17.6%)
Using watches and rings will increase the risk of meat contamination	74 (44.8%)	12 (7.3%)	49 (29.7%)	21 (12.7%)	9 (5.5%)
Hand washing before handling meat reduces the risk of contamination	118 (71.5%)	9 (5.5%)	7 (4.2%)	4 (2.4%)	27 (16.4%)
Regular training could improve meat safety and hygiene practices	103 (62.4%)	38 (23.0%)	4 (2.4%)	4 (2.4%)	16 (9.7%)
Safe meat handling to avoid contamination is part of meat handler job	88 (53.3%)	50 (30.3%)	6 (3.6%)	3 (1.8%)	18 (10.9%)
keeping working surface and utensil clean reduces the risk of illness	116 (70.3%)	4 (2.45)	7 (4.2%)	3 (1.8%)	35 (21.2%)
Surface and equipment should be clean before reusing for meat process	119 (72.1%)	4 (2.4%)	3 (1.8%)	2 (1.2%)	37 (22.4%)
Knives, hooks and cutting boards can be a source of food contamination	79 (47.9%)	14 (8.5%)	47 (28.5%)	5 (3.0%)	20 (12.1%)
knive and cutting boards properly sanitized to prevent cross contamination	131 (79.4%)	2 (1.2%)	1 (0.6%)	2 (1.2%)	29 (17.6%)
Sneezing without covering our noses or mouth could contaminate the meat	86 (52.1%)	16 (9.7%)	38 (23.0%)	14 (8.5%)	11 (6.7%)
Wearing protective clothing and shoes could help improve hygiene practice	130 (78.8%)	17 (10.3%)	1 (0.6%)	2 (1.2%)	15 (9.1%)
Putting on hair cover on the head is a good practice in food industry	110 (66.7%)	24 (14.5%)	3 (1.8%)	2 (1.2%)	26 (15.8%)
**Attitude rank**	**Poor attitude**		19(11.5%)		
	**Fair attitude**		51(30.9%)		
	**Good attitude**		95(57.6)		

**Table 5:** Factors associated with hygienic practices in slaughterhouse and meat retail shops in Hawassa city, Ethiopia, (n = 165).

Variables	Category	Hygienic practice category		COR (95% CI)	P-values	AOR (95% CI)
		Poor practice	Good practice			
Educational status of respondents	No formal education	7	6	1.929(0.558-6.660)	0.486	1.982(0.290-13.566)
	Primary	80	20	0.563(0.261-1.210)	0.869	0.908(0.290-2.844
	Secondary and above	36	16	1	–	1
Attitude	Poor attitude	18	1	0.087(0.011-0.680)	0.056	0.101(0.010-1.057)
	Fair attitude	47	4	0.133(0.044-0.401)	**0.011**	0.157(0.037-0.659)*
	Good attitude	58	37	1	–	1
Knowledge	Insufficient knowledge	73	16	0.421(0.205-0.865)	0.358	1.754(0.530-5.812)
	Good knowledge	50	26	1	–	1
Experience	1-4	57	21	0.632(0.219-1.819)	0.460	0.506(0.083-3.085)
	5-8	54	14	0.444(0.148-1.338)	0.197	0.307(0.051-1.845)
	≥9	12	7	1	–	1
Educational status of meat retail shop owner	Primary	46	11	0.594(0.273-1.294)	0.078	0.300(0.079-1.144)
	Secondary and above	77	31	1	–	1
Regular supervision by health office	Yes	109	35	1	–	1
	No	14	7	0.642(0.240-1.718)	**0.036**	0.176(0.035-0.894)*
Health and safety training	Yes	49	23	1.828(0.902-3.706)	**0.046**	3.46(1.054-11.344)*
	No	74	19	1	–	1
Cleanness and disinfection of Waste containers status	Yes	86	10	7.438(3.316-16.684)	**p < 0.001**	12.64(3.936-40.562)*
	No	37	32	1	–	1
Designate the area where meat stored and ensure restricted entry	Yes	50	22	1	–	1
	No	73	20	1.606(0.794-3.248)	0.372	0.602(0.197-835)
Presence of solid and liquid waste management plan.	Yes	19	10	1	–	1
	No	104	32	1.722(0.722-4.051)	0.621	0.716(0.190-2.697)
Removal of personal items during meat processing (behavioral factor)	Yes	72	6	1	–	1
	No	51	36	0.118(0.046-0.301)	**p < 0.001**	0.036(0.008-0.150)*

*Significant at P value<0.05, COR = crude odds ratio, CI = confidence interval, AOR = adjusted odd ratio.

### Environmental hygiene assessment

Approximately half 93 (56.4%) of the meat retail shops lacked the necessary designation to identify the storage area and to ensure restricted entry for others. A total of 57 (34.5%) of the meat retail shops had surfaces that were not smooth and could be cleaned and disinfected. To prevent contamination from the floor, most (159, 96.4%) of the meat from the retail meat stores was kept on hanging. In this study, although some of the meat retail stores had fly screens (5, 3.0%), rat taps (3, 1.8%), and housefly traps (11, 6.7%), the bulk (146, 88.5%) did not have any insect or rodent traps. Most of the study units (55.8%) did not consistently keep their waste containers clean and hygienic. Nonetheless, solid and liquid waste management strategies were present in fewer than half (17.6%) of the meat retail establishments. In slaughterhouses and retail meat stores, hangers were used for around 150 (90.9%) of the meat storage settings.On the other hand, 3.6% of the meat from retail shops was stored in a refrigerator, a freezer, a chilled room, 3.6% was stored in a guarded glass compartment, 0.6% was stored on floors, and 1.2% was stored on a table. Approximately two-thirds (74.5%) of the study units had a potable/safe/adequate water supply in the compound.

### Factors associated with hygienic practices in slaughterhouses and meat retail shops

This study’s bivariate analysis revealed that hygienic practices were linked to the following: the respondents’ educational status, attitude, knowledge, and experience; the owner of the meat retail shop’s educational status; regular health office supervision; health and safety training; the cleanliness and disinfection of waste containers; the designation of the area where the meat was stored and the guarantee of restricted entry; and the existence of solid and liquid waste management plans and Removal of personal items during meat processing (behavioral factor).. The results from multivariable logistic regression revealed that butchers with a fair attitude were 84.3% less likely to practice good hygiene than those with a good attitude [AOR = 0.157; 95% CI: (0.037, 0.659)]. Good hygienic practices were 12.64 times more likely common in meat retail establishments that routinely kept waste containers clean and disinfected than in those that did not [AOR = 12.64; 95% CI (3.936--40.562)]. Meat retail shops that were not regularly supervised by health offices were 82.4% less likely to practice good hygiene than those were [AOR = 0.176; 95% CI: (0.35–0.894)]. on the other hand, men who had received any kind of health and safety training were 3.46 times more likely to practice good hygienic practices than those who had not [AOR = 3.46; 95% CI (1.054–11.344)]. Furthermore, butcher man who keep personal items during meat processing were about 96.4% less likely to have good hygienic practice than those who remove them [AOR = 0.036; 95% CI (0.008–0.150)} (Table 5).

### Observational assessment result for meat retail shops

All 141 (100%) of the meat retail shops get their meat products from the Hawassa City municipality slaughterhouse, and 141 (100%) of them have approved sources. In addition, 141 (100%) of meat retail shops had stamps on the carcasses from authorized institutions. Below the half, 41 (25%) of meat retail shops were found to have poor construction quality, inadequate ventilation, and no natural or artificial light. In addition, 132 (80%) of them do not restrict the entrance of insects, birds, rodents, and other vermin to prevent the entry of environmental contaminants and cross-contaminants. More than half, 124 (75%), of the units were laid out and equipped in a manner so as to ensure that edible meat does not come into contact with floors, walls, or other fixed structures, and also 91 (55%) of the meat retail shops’ unit floors and walls are not waterproof, non-absorbent, and washable, and the floors of the unit are not non-slippery. More than half, 115 (70%), of meat retail shops’ ceilings and roofs are not so constructed and finished so as to minimize condensation, mold development, flaking, and accumulation of dirt. Also, 148 (90%) of the unit’s windows and other openings are not so constructed so as to avoid the accumulation of dirt and are not fitted with insect screens. None of them had a supply of hot potable water available during working hours. The majority, 157 (95%), of meat retail shops had no adequate and conveniently located facilities for handwashing. 115 (70%) of meat retail shops’ equipment and utensils used in the establishment have a smooth impervious surface and are resistant to corrosion. In addition, 99 (60%) of the units had no efficient effluent and waste disposal systems. In 157 (95%) of meat retail shops, there is no effective and continuous pest control program for controlling insects, birds, rodents, or other vermin. However, 115 (70%) of the meat handlers at the unit are medically examined prior to their employment for infectious and communicable diseases. Although 132 (80%) of the meat handlers did not maintain a high degree of personal cleanliness, wash their hands frequently, or wear clean aprons, head covers, and footwear. More than half, 107 (65%), of meat retail shops had adequate facilities for the storage of raw meat and cooked meat. Below the half percent 50 (30%) of cold storage was not clean. However, 91 (55%) of meat retail shops properly stored meat, and the majority, 157 (95%), of meat retail shops do not maintain temperature, and all refrigerated spaces are not equipped with temperature measurement or recording devices (Table 6).

**Table 6:** Observational assessment result summary for meat retail shops in Hawassa city, Ethiopia.

Question items	Response (n = 141)	N (%)
Source of meat supply with the name and address of supplier	municipality slaughterhouse	141 (100%)
	In the butcher house	0(0%)
Whether approved or unregistered	Approved	141 (100%)
	Unapproved	0 (.0%)
Whether there is stamp on the carcasses from authorized institution	Yes	141 (100%)
	No	0(0%)
Construction is sound having adequate ventilation, good natural or artificial light	Yes	124(75.0%)
	No	41(25.0%)
Restrict the entrance of insects, birds, rodents, and other vermin	Yes	33(20.0%)
	No	132(80.0%)
The unit is laid out and equipped in a manner so as to ensure that edible meat does not come in to contact with floors, walls or other fixed structures	Yes	124(75.0%)
	No	41(25.0%)
Floors and walls are waterproof, non-absorbent, and washable and the floors of the unit are non-slippery	Yes	74(45.0%)
	No	91(55.0%)
Ceiling/ roofs are so constructed and finished so as to minimize condensation, mold development, flaking and accumulation of dirt	Yes	50(30.0%)
	No	115(70.0%)
Windows and other openings so constructed so as to avoid accumulation of dirt and fitted with insect screen	Yes	17(10.0%)
	No	148(90.0%)
Supply of hot potable water is available during working hours	Yes	0(0%)
	No	165(100.0%)
Adequate and conveniently located facilities are provided for hand wash	Yes	8(5.0%)
	No	157(95.0%)
Equipment and utensils used in the establishment have smooth impervious surface, are resistant to corrosion	Yes	116(70.0%)
	No	49(30.0%)
The unit has an efficient effluent and waste disposal system	Yes	66(40.0%)
	No	99(60.0%)
There is an effective and continuous pest control program.	Yes	8(5.0%)
	No	157(95.0%)
The meat handlers at the unit are medically examined prior to their employment	Yes	16(80.0%)
	No	4(20.0%)
The meat handlers maintain a high degree of personnel cleanliness	Yes	33(20.0%)
	No	132(80.0%)
Adequate facilities exist for storage of raw meat and cooked meat	Yes	107(65.0%)
	No	58(35.0%)
Conditions of cold storage clean	Yes	116(70.0%)
	No	49(30.0%)
Meat properly stored	Yes	91(55.0%)
	No	74(45.0%)
Temperature is maintained or not whether all refrigerated spaces are equipped with temperature measurement or recording devices	Yes	8(5.0%)
	No	157(95.0%)

### Observational assessment result for slaughterhouse

The general premise was that the slaughterhouse was ideally not linked to a meat market located away from vegetable, fish, or other food markets. The condition of the lairage/holding pens was hygienic and adequate for the processing plant. Sufficient precautions and care were not taken to ensure that the animals being slaughtered did not have access to see the other ones being slaughtered. The floors in the building were not made of impervious and non-slippery materials. The ceilings and roofs were not constructed and finished so as to minimize condensation, mold development, flaking, and accumulation of dirt. The windows, doors, and other openings were not fly-proof. An adequate and efficient drainage system exists for the washing of animals in lairage and cleaning of carcasses in the slaughterhouse, and all drains are properly installed. There was no proper drainage system for blood and no arrangements to prevent the entry of scavengers like rats, mice, and vermin. There were adequate facilities and equipment and a supply of clean, potable cold water with pressure hose pipes, but there was no supply of hot water available for slaughter according to production capacity. The slaughterhouse waste was disposed of. Animals were not rendered unconscious or stunned before slaughtering was done, and there was no method of stunning used. There were no hygienic conditions during slaughtering; precautions were not taken to avoid contamination and cross-contamination. The workers were not neat, clean, and tidy and were not provided with proper clean aprons and headcovers.

### Laboratory result for slaughterhouse

Table 7 shows the 100% presence of indicator bacteria, i.e., E. coli, in colony-forming units from all sample sources of the slaughterhouse. The mean bacterial load in the hand swab category was 60.6*10^2^ CFU/ml. Although the standard deviation was 22.17*10^2^ CFU/ml., this suggests relatively low variability in hand hygiene. The lower variability may suggest some consistency in hand hygiene, though the contamination level was still concerning. Hands of meat processors were contaminated, but less so compared to the cloth and cutting board swab test category. The maximum bacterial load in the hand swab category was 99*10^2^ CFU/ml. Although the mean bacterial load in the cutting board was 103.3*10^2^ CFU/ml. The standard deviation was 69.99*10^2^ CFU/ml, indicating considerable variability. Cutting boards show a high contamination level, though lower than cloths. The wide standard deviation suggests inconsistent cleaning practices. Some cutting boards may be cleaned properly, while others are significantly contaminated. The maximum bacterial load counted in the cutting swab category was 233*10^2^ CFU/ml. Inaddition, the mean bacterial load in the cloth category was 201.2*10^2^ CFU/ml, while the standard deviation was 53.99*10^2^ CFU/ml, indicating moderate variability in contamination levels across cloth swab samples. Meat processors’ clothes were the most contaminated surfaces on average, and variation suggests that while some meat processors’ clothes were contaminated, most have relatively consistent high contamination. This indicates that meat processors’ clothes were likely not cleaned effectively or frequently enough. The maximum bacterial load count in the cloth swab category was 274.30*10^2^CFU/ml. Furthermore, the mean bacterial load in the knife swab sample was 50.6*10^2^ CFU/ml, while the standard deviation was 29.44*10^2^ CFU/ml, showing moderate variability. Knives, while less contaminated on average, still pose a hygiene concern. The variability suggests that some knives may be effectively cleaned, while others remain heavily contaminated. The highest bacterial count load in the knife swab category was 86*10^2^ CFU/ml.

**Table 7:** Microbial results of meat handlers and utensils in slaughterhouse in Hawassa city, Ethiopia.

Descriptive Statistics					
	N	Minimum (CFU/ml)	Maximum (CFU/ml)	Mean (CFU/ml)	Std. Deviation (CFU/ml)
hand swab	10	33*10^2^	99*10^2^	60.6*10^2^	22.17*10^2^
cutting board	10	25*10^2^	233*10^2^	103.3*10^2^	69.99*10^2^
cloth	10	132*10^2^	274.30*10^2^	201.2*10^2^	53.99*10^2^
knife	10	10*10^2^	86*10^2^	50.6*10^2^	29.44*10^2^

### Laboratory swab test result for meat retail shop

The presence and absence of indicator bacteria for sanitary quality, i.e., E. coli, in all sample sources, with minimum and maximum ranges and the frequency and percentages in the meat retail shops, were presented in Tables 8 and 9, respectively. Since each of the 141 swab samples was measured in four different categories, the total number of observations became 564. From the hand swab category, 100 (70.92%) of the samples had *E. coli* with a mean average of (66.54 ± 63.8)*10^2^ CFU/ml. Hands of meat handlers had a lower average contamination level compared to cloths and cutting boards, but were still significantly contaminated. There is substantial variability in hand cleanliness among meat handlers. Hence, the highest bacterial count or contamination on hand was 196*10^2^CFU/ml, suggesting that some workers might not be practicing proper hand hygiene. Similarly, in a cloth swab sample category, the presence of indicator bacteria was 106 (75.18%), with a count of (76.93 ± 70.18)*10^2^ CFU/ml from the total sample. There is a large variation in contamination levels across cloth swab samples, indicating some areas may have very high contamination, while others have low or no contamination. The highest contamination detected on the cloth swab was 271 CFU/ml. 103 (73.05%) of swab samples from the knives category were positive for E. coli, with a mean average count of (68.37 ± 62.74)*10^2^ CFU/ml. Inaddition, Knives show moderate average contamination, higher than hand swabs but lower than cutting boards, and although the standard deviation value shows there is a wide variation in contamination levels on knives among meat retail shops. Some knives had high contamination levels with a count of 198 CFU/ml, which could indicate poor cleaning between uses. Furthermore, 110 (70.01%) of the cutting board swab sample category were positive for E. coli, with a mean average count of (78.72 ± 72.69)*10^2^ CFU/ml. Cutting boards had the highest average contamination across all categories. There is also a wide variability in contamination levels among all cutting board swab categories. Hence, the highest contamination level was observed on the cutting board, i.e., 285 CFU/ml, indicating they may pose a higher food safety risk if not cleaned properly. Overall, the total of the swab sample tests indicates that from the total of 564 observations, 145 (25.71%) show the absence of sanitary indicator bacteria (E. coli), and 419 (74.29%) samples show the presence of sanitary indicator bacteria (E. coli). All swabbed sample items showed considerable bacterial contamination. There is also significant variability in cleanliness across all categories, suggesting inconsistent hygiene and sanitation practices among meat retail shops. (Table 8).

**Table 8:** Microbial load results of meat handlers and utensils in a meat retail shop in Hawassa city, Ethiopia.

Descriptive Statistics					
	N	Minimum (CFU/ml)	Maximum (CFU/ml)	Mean (CFU/ml)	Std. Deviation (CFU/ml)
Hand swab	141	0	196*10^2^	66.54*10^2^	63.8*10^2^
Swab from cloth	141	0	271*10^2^	76.93*10^2^	70.18*10^2^
Swab from knife	141	0	198*10^2^	68.37*10^2^	62.74*10^2^
Swab from cutting board	141	0	285*10^2^	78.72*10^2^	72.69*10^2^

**Table 9:** Microbial results: presence and absence percentages of meat handlers and utensils in meat retail shop in Hawassa city, Ethiopia.

Hand swab category		N(%)
	≤ 0	41(29.08%)
	≥1	100(70.92%)
	Total	141(100.0%)
**Cloth swab category**		
	≤ 0	35(24.82%)
	≥1	106(75.18%)
	Total	141(100.0%)
**Knife category**		
	≤ 0	38(26.95%)
	≥1	103(73.05%)
	Total	141(100.0%)
**Cutting board category**		
	≤ 0	31(29.99%)
	≥1	110(70.01%)
	Total	141(100.0%)
**Overall total**		
	≤ 0	145(25.71%)
	≥1	419(74.29%)
	Total	564(100%)

≤ 0 = absence of E.coli, ≥1= presence of E.coli.

## Discussion

In this study, the prevalence of good hygienic practices was 25.5% (95% CI: 18.2–32.1). The findings of this study are lower than those of studies conducted in Nigeria [23], Chitwan, Nepal [61], Kathmandu, Nepal [62] and Gonder, Ethiopia [25]. However, the prevalence of good hygienic practices in the present study was higher than that reported in studies conducted in Jigjiga, Ethiopia, [56]. This discrepancy might be due to the fact that awareness, attitudes, or lack of essential facilities such as sanitation and hygiene facilities do not make practice as expected in developed countries like Ethiopia. The present study revealed that the attitudes of meat handlers, the cleanliness and disinfection status of waste containers, regular supervision by health offices, and health and safety training are the main factors influencing the poor hygienic practices of meat handlers. However, improper handling and unsafe hygiene inevitably lead to the contamination of meat, eventually affecting the health of consumers [63]. In the present study, attitudes and hygienic practice levels were inconsistent. This may be attributed to either butchers’ negligence in daily practices or inefficient supervision from the concerned authorities.

Personal hygiene practices are critical for ensuring food safety and protecting consumers from food-borne infections and intoxication. In the present study, all of the butcher men practiced washing their hands with soap and water after visiting the toilet. This is in contrast with a study conducted in Jigjiga, Ethiopia, where 86.8% of respondents reported always washing their hands after using the toilet [56]. This percentage than the 75.4% reported in Malaysian canteens [64]. The high prevalence of handwashing practices in the present study may be influenced by the self-reported nature of the data, which can sometimes lead to respondents overreporting positive behaviors to present themselves positively, such as handwashing during meat processing. Maybe butchers in the present study had better access to hygiene education or food safety training. According to the CAC – Codex Alimentarius Commission [65], improper food handling and poor hand hygiene are the main risk factors for the occurrence of food contamination that leads to food-borne diseases. In the present study, 89.7% of the respondents did not wash their hands after they handled waste or garbage. Moreover, 37% of meat handlers did not wash their hands after smoking, sneezing, or coughing. The codex recommended that meat handlers wash their hands at all stages of food production to protect consumers from diarrhea and other food-borne diseases before handling meat; after eating, smoking, coughing, sneezing, touching garbage, and using the toilet are critical times when the meat handler should wash their hands. Meat handlers with open skin injuries, gastroenteritis, and ear or throat diseases should not handle any meat production [65].

Attitude has the most powerful influence on food workers’ degree of practice. In this study, significant associations were observed in the practice of meat hygiene attitudes (fair attitudes). Butchers who had a fair attitude were 84.3% less likely to practice good hygiene than those who had a good attitude. A total of 57.6% of butchers had good attitudes toward food safety, 30.9% had fair attitudes, while the remaining 11.5% had poor attitudes toward food safety. This finding agrees with study conducted in Nigeria, approximately half of the respondents (53.1%) had good attitudes toward meat hygiene, 36.5% had fair attitudes, and 10.4% of the respondents had poor attitudes toward meat hygiene [23]. Moreover, approximately 64% of respondents had good attitudes toward food safety, according to research done in Jigjiga, Ethiopia [56]. Meat handler attitudes play an important role in influencing food safety practices, which helps reduce the likelihood of food-borne disease outbreaks. Akabanda et al. [60] reported a strong link between positive attitudes and maintaining safe food handling practices.

In this study, meat retail shops that consistently maintained cleanliness and disinfected their waste containers were 12.64 times more likely to follow good hygienic practices compared to those that did not. In this study, 58.2% of the meat retail shops did not regularly maintain the cleanliness and disinfection status of their waste containers. Moreover, the cleanliness and disinfection of waste containers were significantly associated with the hygienic practices of meat handlers. This may be due to the negligence of cleaners or meat handlers. This process is also a major contributor to the residence or multiplication of different microorganisms (bacteria) inside the waste container. This hygiene and sanitation facility is an environmental factor that can contaminate meat with microorganisms when meat handlers act as mechanical transmitters from waste containers to meat when they are in contact with their hands or clothes. Cleaning and disinfecting beef retail shops on a regular basis are vital since they help reduce microbial contamination. According to an observational checklist, the majority of butcher shops are situated on the road’s edge, where they are subject to wind or vehicles induced dust which might contaminate them organisms. Most of the butcher shops examined had poor hygienic cleaning standards. A total of 91.5% of the respondents did not properly clean the meat storage area before storing new products. Only 45.5% of the respondents claimed to use disinfectants when washing. Additionaly, 92.1% of participants did not replace or sterilize their knives after each meat processing. These findings algin with Ali et al’s study in Karachi, Pakistan [66], which reported a lack of knowledge of disinfection and sanitization by butcher men. Similarly, a study conducted in Ghana; found that 100% of meat sellers did not disinfect their shops, and 95% did not sterilize their knives and equipment, but The few who sterilized their knives and equipment used hot water, according to Adzitey [67]. This is also in agreement with a study conducted in Jimma, Ethiopia; where none of the participants washed or disinfected their processing tools or floors after each working interval. The same cutting boards and knives were used for the cutting of meat and abdominal organs [21].

The present study revealed that meat retail shops without regular supervision from health offices were 82.4% less likely to practice good hygiene compared to those with regular supervision. Approximately 87% of the meat retail shops in the study were regularly supervised by a health office. In contrast, Jeffer et al (38). reported that government oversight of butcher shops in Uganda was very poor, with only 8% of butcher shops reporting onsite inspections by government authorities and only 2% being aware of the government’s specific requirements for operating meat retail shops [55]. This could be one reason behind the poor practice of hygiene in the present study according to the statistical findings. The presence of regulatory authority is important in maintaining good hygiene practices through the implementation of rules and regulations, which have good hygienic effects on meat handling standards. These rules and regulations regarding good hygiene are to be respected by meat retail shops to maintain good hygiene, and these regulatory bodies teach appropriate meat handling practice standards and appropriate measures if the meat handling standards are violated. The statistical association reveals how significant regular supervision is in controlling or managing good hygienic practices. However, even in the presence of regular supervision by health offices, good hygienic practices may not be implemented in some meat retail shops. This may be due to health behavior problems and the negligence of meat handlers and owners of meat retail shops.

Inaddition, men who had received health and safety training were 3.46 times more likely to practice good hygiene than those who had not received such training. This implies that health safety and training are important for improving the hygienic practices of meat handlers. The high levels of inadequate sanitation procedures observed among meat handlers could be attributed to a lack of training. According to Adams and Moss (48), training food handlers regarding the basic concepts and requirements of personal hygiene plays an integral role in ensuring safe products for consumers [68].

Furthermore, butchers who kept personal items during meat processing were about 96.4% less likely to have good hygienic practice than those who removed them [AOR = 0.036; 95% CI (0.008–0.150)}. A study conducted in the Gedeo zone, southern Ethiopia, found 51.3% of meat handlers did not remove rings, necklaces, or watches during meat handling; thus, the practices linked to poorer hygiene knowledge and practices support the negative impact of personal items on hygiene. [69]. Another study conducted in North Shewa Zone, Oromia, Ethiopia, indicates 51.3% had good meat handling practices, but personal items were not directly analyzed by the Adjusted Odds Ratio (AOR). The common observation was that the removal of personal items improved hygiene. [70]. In addition, a study conducted in Gonder town, Ethiopia, revealed good meat handling practices not directly analyzed by the Adjusted Odds Ratio (AOR) for personal items. Observationally, wearing personal items correlated with poorer hygiene [25]. This study indicates a strong negative association far more extreme than observational findings from other Ethiopian contexts. However, the direction is consistent: removing personal items improves hygiene. In addition, the studies show personal item use correlates with poorer practice. This implies the current study’s finding provides quantitative strength to existing observational trends in Ethiopia. This encourages formal regulation and training, emphasizing the removal of personal items during meat handling practices to prevent cross-contamination.

Although there was supportive evidence to confirm the hygienic practice, if it is poor or good in the slaughterhouses and in meat retail shops, in addition to the questionnaire survey and observation checklis result. The laboratory test result shows 100% presence of indicator bacteria, i.e., *E. coli*, in colony-forming units from all sample sources of the slaughterhouse. The presence of *E. coli* in all samples tells us of the poor sanitary quality. The majority of *E. coli* strains do not cause disease and are part of the normal flora of animals’ and humans’ intestinal tracts, but the presence of *E. coli* in foods intended for human consumption indicates poor sanitary and hygiene practices during production, processing, transportation, or preparation [20]. In the present study, of a total of 604 samples taken from slaughterhouse and meat retail shops, 459 (75.99%) were confirmed as positive for *E. coli*. With regard to sample sources, 40 (100%) of *E. coli* were detected in slaughterhouse samples, and 419 (74.29%) were detected in meat retail shop samples. This is in contrast with a study conducted in Jimma town where, of a total of 505 samples, 102 (20.2%) were confirmed as positive for *E. coli*. With regard to sample sources, 55 (19.3%) of *E. coli* were detected in abattoir house samples, and 47 (21.4%) were detected in butcher shop samples, respectively [21]. In the present study, there was high E. coli. The discrepancy between the two studies might be the difference in the level of poor hygienic practice. The current study suggests significantly poorer sanitary conditions. Jimma’s lower contamination may reflect better cleaning protocols, trained personnel, or regulated oversight. Lack of regular inspection or weak enforcement mechanisms might be more pronounced in the study area. Overall, the total of the swab sample tests from meat retail shops indicates that from the total of 564 observations, 145 (25.71%) show the absence of sanitary indicator bacteria (E. coli), and 419 (74.29%) samples show the presence of sanitary indicator bacteria (E. coli). All swabbed sample items showed considerable bacterial contamination. There is also significant variability in cleanliness across all categories, suggesting inconsistent hygiene and sanitation practices among meat retail shops. According to Codex Alimentarius (FAO/WHO) codes of practice, food contact surfaces, equipment, and personal clothing should be free of E. coli. *E. coli* presence is considered a hygiene indicator organism. Hence, detection of *E. coli* suggests inadequate sanitation or contamination. [71]. Hence, this study’s meat contact surface swab sample analysis and checklist observation results highlight the urgent need for better cleaning procedures, regular monitoring, and improved personal hygiene practices in the meat-handling environment, both in the meat retail shops and the municipal slaughterhouse.

### Limitations of the study

This study shares the limitation of a cross-sectional study design, which is unable to establish a cause and effect relationship and demonstrates the presence of an observable relationship between exposure and consequence at the same time.

## Conclusion

This study revealed poor hygienic practices in meat handlers. This may have implications for the cross-contamination of meat. After adjusting for possible confounding factors, attitudes, cleanness and disinfection of waste containers, regular supervision by health offices, health and safety training, and removal of personal items during meat processing (behavioral factor) were significantly associated with hygienic practices in slaughterhouses and meat retail shops.

The government should create abattoir policy guidelines to achieve near-perfect hygiene. They should also provide butchers with safe clothing, such as masks, boots, and aprons, as well as free formal hygiene training. The government should construct a modern abattoir for butchers with an adequate bathroom and hand-washing facilities as well as a portable water supply. Municipalities and health offices must regularly supervise slaughterhouses and meat retail shops on the basis of appropriate hygienic practice standards for meat handling checklists to protect public health. Health education programs focusing on behavioral change control and raising community awareness about the importance of butcher hygiene should be devised and implemented. Updates and refresher training should be performed on a more regular basis. This will aid meat handlers in gaining a better awareness of the dangers connected with potential disease contamination as well as cleanliness practices. This study will provide baseline information for future researchers on hygienic practices in slaughterhouses and meat retail shops, who would like to conduct detailed and comprehensive studies either in Hawassa city or other study areas.

## Supporting information

S1 File Step-by-step protocol. (PDF)

S2 File Data on survey questioner. (XLSX)

S3 File Meat retail shop Swab sample Data. (XLSX)

S4 File Slaughterhouse Swab sample Data. (XLSX)

## Abbreviations

AOR: Adjusted odds ratio
CFU: Colony-forming unit
CI: Confidence interval
COR: Crude odds ratio
*E. coli*: *Escherichia coli*
EHEDG: European Hygiene Equipment Design Group
FMHACA: Food, Medicine and Health Care Administration and Control Authority
IRB: Institutional Review Board
mTSB: modified tryptone soy broth
PPE: Personal Protective Equipment
*S. aureus*: *Staphylococcus aureus*
SPSS: Statistical Package for the Social Sciences
TSI: Triple Sugar Iron
WHO: World Health Organization.
