# Supplementary material for: Hygienic practices and associated factors in slaughterhouses and meat retail shops in Hawasa City, Ethiopia
Source: PLoS One. 2025 Nov 13;20(11):e0336784. doi: 10.1371/journal.pone.0336784 (PMC12614582; doi:10.1371/journal.pone.0336784)
Supplement: S1 File — (PDF) [file pone.0336784.s001.pdf]

Laboratory procedure (Protocol)

Standardized protocol for surface swab sampling and bacterial load quantification using spread plate and indole confirmation techniques

Funding

The authors did not receive support from any organization for the submitted work.

No funding was received to assist with the preparation of this protocol.

No funding was received for conducting this protocol.

No funds, grants, or other support was received.

Competing interests: The authors declare that they have no competing interests.

Data availability

All data generated using this protocol will be available as supplementary material.

Associated content

This protocol was developed based on established methods adopted from peer-reviewed literature, laboratory manuals, and microbiological guidebooks. All relevant sources are properly cited within the protocol text.

Abstract

This protocol describes a method for surface swab sampling, microbial load quantification, and confirmatory biochemical identification of bacterial contaminants. It includes steps for swab collection, serial dilution, plating, incubation, and the indole test to identify indole-positive organisms. The method is designed to assess hygienic status of surfaces in food handling environments.

Method

A laboratory-based procedure was conducted to assess microbial contamination on food contact surfaces (Hand, cloth, knife, and cutting board) using the swab sampling method. Surface samples were collected from defined areas using sterile cotton swabs, followed by bacterial load quantification through spread plate techniques and confirmatory identification via indole testing.

Materials and Reagents

SWAB TECHNIC

Media and Reagents:

- Buffered peptone water[1]
- Modified Trypone Soya Broth(mTSB)[2,3]
- MacConkey agar[4]
- Indole
- Alcohol
- Distilled Water

So as to process the Swab technique, the materials this should be required

- Cotton swab [1]
- Hot water bath 45°C
- Sterile Petri dishes
- Bunsen burner Flame [5]
- Colony counter with magnifying glass [5]
- Sterile capped 16*150 mm test tubes

- Pipettes of various sizes (e.g. 01, 1.0 and 2.0 mL)[5]
- Balance[5]
- Blender and sterile blender jars[5]
- Incubators at 35 ± 1.0°C[5]
- Manual or Automatic colony counter[5]
- Sterile forceps, spoon, knife, scissors and other sterile sampling equipment[5]
- Transfer loop, 3 mm[5]
- Refrigerator at 2-8°C[5]

Procedure:

1. Determine 100 cm^2 are in order to take surface sample (it could be 10X10 or 20X5)[6]
2. wet the cotton swab using sterile distilled water
3. swab the area

Swabbing procedure

4. Put the swab in to the Buffered peptone water tube.
5. Bend and break the swab as short as possible without touching it [7].

Swab bending procedure

6. Wait 15 min in order to pass all microorganism to the isotonic water [8]
7. Make spread plate inoculation
8. Inoculate 1 day to obtain results. In order to make total mesophilic bacteria count incubate at 37°C

Procedure of Spread Plate Technique

A. Serial Dilution

1. Prepare a series of at least 2 test tubes containing 9 ml of sterile distilled water.
2. Using a sterile pipette, add 1ml of sample in the first tube of the set. *Label it as* 10^{-1}
3. Mix the contents well by swirling the tube upside down few times.
4. From the first tube, take 1ml of the sample and transfer to second tube. *Label it as* 10^{-2} [9]

B. Plating

5. Pipette out 0.1 ml from the appropriate desired dilution series onto the center of the surface of an agar plate.
6. Dip the L-shaped glass spreader *(hockey stick)* into alcohol.
7. Flame the glass spreader over a bunsen burner.
8. Spread the sample evenly over the surface of agar using the sterile glass spreader, carefully rotating the Petri dish underneath at an angle of 45°at the same time.
9. Incubate the plate at 37°C for 24 hours.
10. Calculate the colony forming units (CFU) value of the sample. Once you count the colonies, multiply by the appropriate dilution factor to determine the number of CFU/ml in the original sample[9].

Calculation of result:

$$N = \frac{\mathrm{n}}{s \times d\prime} \text{ [10]}$$

Where N=total number of bacteria (cfu) per ml of the sample, n = average number of bacterial colonies, from different dilutions (10^{-1} - 10^{-3}) in Petri dish that contained 30–300 colonies, s = volume of sample for plating, and d= dilution factor of the specimen/food sample.

For example, suppose the plate of the 10^2 dilutions yielded a count of 130 colonies. Then, the number of bacteria in 1 ml of the original sample can be calculated as follows:

$$\text{Bacteria/ml}(N) = \frac{130\ \text{CFU}}{1ml \times 10^{-2}}$$ = 1.3 × 10^4 CFU/ml or 13,000.

Confirmatory test

Procedure and results for Indole test

Conventional tube method

The main requirement for a suitable indole test medium is that it contains a sufficient amount of tryptophan (Tryptone broth, indole-free peptone water medium, Urea-indole medium, Tryptophan peptone broth, Sulfide-indole motility medium (SIM) ...).[11]

1. Inoculate the tryptophan (or peptone) broth with the organism to be tested and incubate at 37 ° C for 24 to 48 hours[11,12].
2. Add 0.5 ml (5 drops) of Kovác's reagent and shake gently[11]
3. Examine the top layer of liquid after about 1 min[11,12]

Results:

A **positive result** is indicated by the presence of a red or red-violet color in the alcohol layer on the surface of the broth[11,12].

A **negative result** appears in yellow. A variable result may also occur, displaying an orange color as a result. This is due to the presence of skatole, also known as methyl indole or methylated indole, another possible breakdown product of tryptophan[11,12].

Timing

Table 1: Estimated timing for the procedure

Step	Approximate Time
Surface Swabbing	5–10 minutes
Incubation in Buffered Peptone Water	15 minutes
Serial Dilution and Plating	20–30 minutes
Plate Incubation	24 hours
Indole Test Incubation	24–48 hours
Indole Reagent Test	1 minute

Expected Results

- ✓ Quantitative data on total mesophilic bacterial load (CFU/cm² or CFU/mL).
- ✓ Identification of indole-positive organisms such as *E. coli*.

Troubleshooting

Table 2: Troubleshooting common laboratory procedure errors

Issue	Possible Cause	Solution
No colony growth	Incorrect incubation temperature	Ensure 37 °C incubation
Contamination	Non-sterile technique	Use aseptic procedures
No color change in Indole test	Insufficient tryptophan	Use appropriate media

Ethics Statement

Not applicable for in vitro environmental sample testing.

Supporting information

The protocol in PDF format available provided as Supporting Information file 1, with the caption: S1: Step-by-step protocol

Authors' contributions

LD, EM, and AE conceptualized the protocol, performed data analysis and interpretation, and contributed to the initial drafting of the protocol manuscript. All authors contributed to the design or refinement of the laboratory procedures, participated in data interpretation, revised the manuscript critically for important intellectual content, approved the final version to be published, and agreed to be accountable for all aspects of the work.

Reference

1. Abayneh M, Tesfaw G, Woldemichael K, Yohannis M, Abdissa A. Assessment of extended-spectrum β - lactamase (ESBLs) – producing Escherichia coli from minced meat of cattle and swab samples and hygienic status of meat retailer shops in Jimma town , Southwest. 2019;1–8.
2. Fratamico PM, Bagi LK, Abdul-Wakeel A. Detection and Isolation of the “Top Seven” Shiga Toxin-Producing Escherichia coli in Ground Beef: Comparison of RapidFinder Kits to the U.S. Department of Agriculture Microbiology Laboratory Guidebook Method. J Food Prot. 2017 May;80(5):829–36.

3. United States Department of Agriculture Food Safety and Inspection Service. Detection, Isolation, and Identification of Top Seven Shiga Toxin-Producing Escherichia coli (STEC) from Meat Products, Carcass, and Environmental Sponges. In: Laboratory Guidebook MLG 5C04 [Internet]. 2024. p. 1–18. Available from: https://www.fsis.usda.gov/sites/default/files/media_file/documents/MLG-5C.04.pdf

4. M. RA. Handbook of Microbiological Media [Internet]. 4th Editio. 2010. Available from: https://doi.org/10.1201/EBK1439804063

5. United States Department of Agriculture Food Safety And Inspection Service O of PHS. Quantitative analysis of bacteria in foods as sanitary indicators effective. In: Laboratory Guidebook Notice of Change Chapter new, revised, or archived: MLG 302 [Internet]. 2015. p. 1–19. Available from: https://www.fsis.usda.gov/sites/default/files/media_file/2021-03/MLG-3.pdf

6. Andrews AWH, Hammack TS. BAM : Food Sampling / Preparation of Sample Homogenate Food Sampling and Preparation of Sample Homogenate. In: Bacteriological Analytical Manual Chapter 1 Food Sampling and Preparation of Sample Homogenate. 2020. p. 1–11.

7. Mooijman KA, Pielaat A, Kuijpers AFA. Validation of EN ISO 6579-1 - Microbiology of the food chain - Horizontal method for the detection, enumeration and serotyping of Salmonella - Part 1 detection of Salmonella spp. Int J Food Microbiol. 2019 Jan;288:3–12.

8. ICMSF. Organisms in foods2 Sampling for microbiological analysis : Principles and specific applications. In: PART II SPECIFIC PROPOSALS FOR SAMPLING AND SAMPLING PLANS [Internet]. Second edi. 1986. p. 129–245. Available from: https://seafood.oregonstate.edu/sites/agscid7/files/snic/sampling-for-microbiological-analysis-principles-and-specific-applications-icmsf.pdf

9. Cappuccino JG, Welsh CT. Microbiology: A Laboratory Manual, Global Edition [Internet]. Pearson Education; 2017. Available from: https://books.google.com.et/books?id=AJIZDgAAQBAJ

10. Atlabachew T, Mamo J. Microbiological Quality of Meat and Swabs from Contact Surface in Butcher Shops in Debre Berhan, Ethiopia. J Food Qual. 2021;2021.

11. Macwilliams MP. Indole Test Protocol [Internet]. 2016. Available from: https://asm.org/getattachment/200d3f34-c75e-4072-a7e6-df912c792f62/indole-test-protocol-3202.pdf

12. Cheesbrough M. District Laboratory Practice in Tropical Countries [Internet]. 2nd ed. Cambridge University Press; 2006. Available from: https://www.cambridge.org/core/books/district-laboratory-practice-in-tropical-countries/6F6FE267756B004A4826209C692BD5B3
